# Bile acid signaling: from dynamic pool composition to inter-organ communication

**DOI:** 10.3389/fendo.2026.1890710

**Published:** 2026-07-24

**Authors:** Fuyu Yang, Ting Wei, Kai Liu, Huikuan Chu, Ling Yang

**Affiliations:** Division of Gastroenterology, Union Hospital, Tongji Medical College, Huazhong University of Science and Technology, Wuhan, China

**Keywords:** bile acids (BAs), Farnesoid X receptor (FXR), gut microbiota, systemic homeostasis, Takeda G-protein receptor 5 (TGR5)

## Abstract

Bile acids (BAs) are synthesized in the liver and extensively modified by gut microbiota in the intestine. The dynamic composition of the BA pool enables BAs to modulate metabolic responses at varying degrees. By activating nuclear (e.g., FXR) and membrane (e.g., TGR5) receptors, BAs integrate metabolic and immune processes across the enterohepatic circulation and peripheral tissues, forming a systemic homeostatic network. Dysregulation of this network is causally linked to metabolic dysfunction-associated steatotic liver disease (MASLD), type 2 diabetes mellitus, and other disorders. Targeting BA signaling has shown therapeutic potential in preclinical studies, though clinical translation requires further exploration. This review elucidates the dynamic composition of the BA pool, the integrated regulatory roles of BA signaling networks in health and disease, and highlights emerging therapeutic strategies that target the BA signaling—including FXR agonists, TGR5 modulators, and microbiota-derived BA interventions—for metabolic and immune-mediated diseases.

## 1 Introduction

Bile acids (BAs) are metabolites whose levels and composition are co-regulated by the host and the gut microbiota. Based on their biosynthetic origin, they are classified into two types: primary BAs, synthesized *de novo* from cholesterol in the liver, and secondary BAs, which are formed from microbial enzymatic modification of primary BAs in the intestine (1). Since the 1990s, the identification of BA receptors (e.g., Farnesoid X Receptor [FXR] and Takeda G-protein receptor 5 [TGR5]) has shifted the perception of BAs from simple digestive detergents to true hormonal molecules (2, 3). Meanwhile, with the deepening understanding of the gut microbiota and its metabolic interactions with the host, a growing diversity of BA modifications and their biological functions have been revealed, including acetylation (4), succinylation (5), and conjugation with alternative amino acids (6), long-chain fatty acids (7), or polyamines (8, 9). Now, the actions of BAs are no longer confined to the gut-liver axis; they also influence systemic metabolism and immune regulation by activating diverse membrane and nuclear receptors (10). Therefore, deciphering the dynamic composition of the BA pool and the BA-mediated signaling networks across different organs is essential for understanding their role in physiology and diseases.

Accumulating evidence indicates that patients with metabolic diseases exhibit disturbed BA metabolism. For example, patients with metabolic dysfunction-associated steatotic liver disease (MASLD) often show abnormal BA metabolism, including elevated total BA levels and altered secondary BA composition (11). In patients with type 2 diabetes mellitus (T2DM), the levels of deoxycholic acid (DCA), lithocholic acid (LCA), and glycodeoxycholic acid (GDCA) are increased, while the level of glycoursodeoxycholic acid (GUDCA) is decreased (12). As the regulatory roles of BA signaling networks in different organs are increasingly defined, these networks have emerged as key players in the pathogenesis of metabolic and immune diseases. Although targeting BA signaling pathways has opened new avenues for the treatment of metabolic and immune diseases, its clinical application still faces considerable challenges and requires further exploration. Thus, this review aims to systematically elucidate the dynamic composition of the BA pool, the integrated regulatory roles of BA signaling networks in health and disease, and the therapeutic interventions targeting BA signaling.

## 2 Dynamic composition of bile acid pool

**Figure 1:**
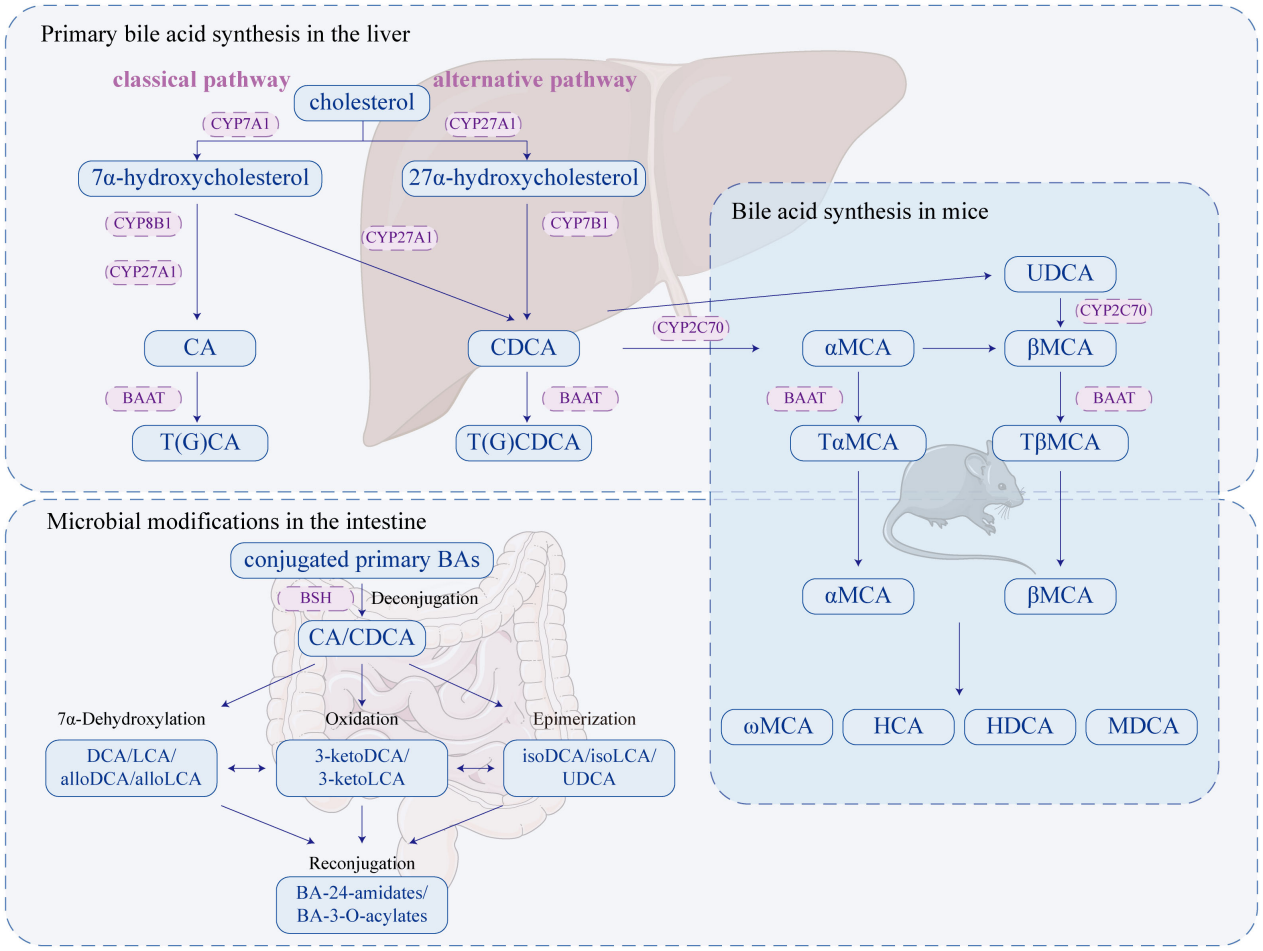
Bile acid synthesis and metabolism. Schematic illustration of the synthetic pathways for primary BAs in hepatocytes (upper left) and secondary BAs in the intestine (lower left). The inset on the right highlights murine BA species that differ from those in humans. BAs, bile acids; BAAT, bile acid CoA:amino acid N-acyltransferase; BSH, bile salt hydrolase; CA, cholic acid; CDCA, chenodeoxycholic acid; CYP7A1, cholesterol 7α-hydroxylase; CYP8B1, cholesterol 12α-hydroxylase; CYP27A1, cholesterol 27-hydroxylase; CYP7B1, cholesterol 7α-hydroxylase; CYP2C70, cholesterol 6β-hydroxylase; DCA, deoxycholic acid; HCA, hyocholic acid; HDCA, hyodeoxycholic acid; MCA, muricholic acid; MDCA, murideoxycholic acid; LCA, lithocholic acid; G, glycine-conjugated species; T, taurine-conjugated species.

BA synthesis represents the predominant pathway for cholesterol catabolism (13). In the adult liver, approximately 500 mg of cholesterol is metabolized into BAs daily through the classic (neutral) and alternative (acidic) pathways (**Figure 1**). The former begins with the rate-limiting enzyme cholesterol 7α-hydroxylase (CYP7A1) to generate cholic acid (CA) and chenodeoxycholic acid (CDCA) (1, 13). Occurring in the liver and other organs (e.g., brain and adrenal glands), the alternative pathway begins with cholesterol 27-hydroxylase (CYP27A1) and subsequently involves the rate-limiting hydroxylation of the intermediate metabolite by CYP7B1 to form CDCA (10, 14). Before being transported out of hepatocytes, BAs undergo a conjugation reaction with glycine or taurine by BA-coenzyme A (CoA) synthetase and BA CoA:amino acid N-acyltransferase (BAAT) (13, 15). Following their exposure in the intestinal tract, BAs undergo extensive modifications by gut microbiota, including deconjugation, 7α-dehydroxylation, oxidation, epimerization and reconjugation (16). These pathways produce more hydrophobic BAs, such as DCA from CA and LCA from CDCA (13). Therefore, the BA pool is a dynamic product shaped by both host synthesis and microbial modification.

Notably, differences exist in BA profiles between humans and rodents (**Figure 1**). In humans, CDCA and CA are the predominant forms, accounting for approximately 80% of the total BA pool; whereas in mice, most CDCA is rapidly metabolized into more hydrophilic muricholic acids (α-MCA and β-MCA), comprising roughly half of their total BA pool (17, 18). Furthermore, in humans, BAs primarily conjugated with glycine; while in mice, they are almost conjugated with taurine (15). Regarding the metabolic capabilities of gut microbiota, the human profile is comparable to that of rats (19). In detail, humans outperform mice in deconjugation and 7α‑dehydroxylation, whereas they are inferior to both rats and mice in oxidation and epimerization (19). These species‑dependent differences in BA composition therefore represent a significant translational hurdle that should be carefully considered when extrapolating findings from animal models to clinical settings.

**Figure 2:**
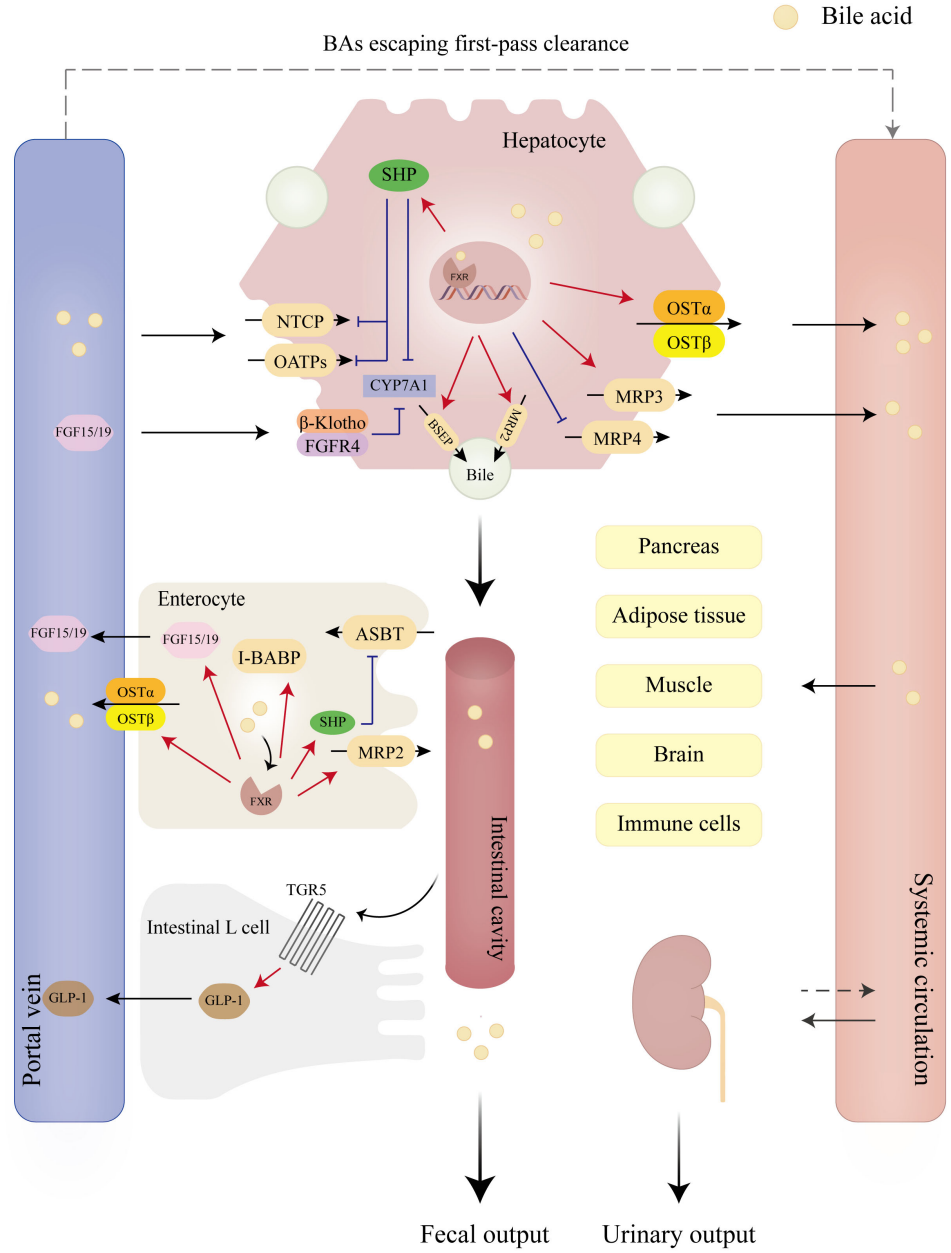
Bile acid metabolic cycle. This diagram systematically illustrates the processes of BA synthesis, enterohepatic circulation, inter-organ transport and excretion in the body. In the liver, BAs are synthesized via CYP7A1 and secreted into bile through BSEP and MRP2; BA synthesis and transports are precisely regulated via FXR-SHP and FGF19-FGFR4 signaling pathway. In enterocytes, BAs are actively taken up via ASBT and finally reabsorbed into the blood via the OSTα/β complex. BAs returning via the portal vein can be taken up into hepatocytes through NTCP and OATPs; some BAs that escape the first-pass clearance enter the systemic circulation. ASBT, apical sodium-dependent bile acid transporter; BAs, bile acids; BSEP, bile salt export pump; CYP7A1, cholesterol 7α-hydroxylase; FGF, fibroblast growth factor; FGFR4, fibroblast growth factor receptor 4; FXR, Farnesoid X receptor; GLP-1, glucagon-like peptide-1; I-BABP, ileal bile acid binding protein; MRP, multidrug resistance-associated protein; NTCP, Na^+^-dependent taurocholate co-transporting polypeptide; OATP, organic anion transporting polypeptide; OST, organic solute transporter; SHP, small heterodimer partner; TGR5, G protein-coupled receptor Takeda G-protein receptor 5.

The enterohepatic circulation of BAs involves two primary processes: hepatic secretion and intestinal absorption (**Figure 2**). In the liver, BAs are secreted into bile primarily via the bile salt export pump (BSEP/ABCB11) and multidrug resistance-associated protein 2 (MRP2) (20). In the ileum, BAs are actively absorbed through the apical sodium-dependent bile acid transporter (ASBT), then shuttled intracellularly by the cytosolic ileal bile acid binding protein (I-BABP) and ultimately transported into the portal blood via the heteromeric organic solute transporter α/β (OSTα/OSTβ) (21). Notably, some BAs fail to be reabsorbed in the ileum; instead, they are transformed by gut microbiota in the colon, and later absorbed via passive diffusion (22). Upon returning to the liver through the portal vein, BAs are taken up into hepatocytes mainly via the Na^+^-dependent taurocholate co-transporting polypeptide (NTCP/SLC10A1) and organic anion transporting polypeptides (OATPs/SLCOs) (14). The liver then recombines these recycled BAs and secretes them back into bile to maintain the cycle.

The composition of the BA pool is subject to real-time regulation by multiple levels of factors. Substantial evidence highlights dietary intake as a key modulator of BA profiles. In a case-control study, patients with dyslipidemia exhibited gut microbiota dysbiosis and marked alterations in their BA profile, including elevated levels of apocholic acid and allocholic acid (23). Consistently, a high-fat diet intervention in a rat model led to significantly increased concentrations of free BAs, primary BAs, and total BAs (23). In a clinical study involving 138 patients, protein intake was positively correlated with the levels of secondary BAs (DCA and LCA), whereas monounsaturated fatty acids (MUFAs) showed a negative correlation with DCA derivatives (24). Moreover, higher intakes of dietary fiber and coffee were positively associated with the proportion of BAs converted to derivatives, potentially promoting the generation of protective metabolites (24). Alcohol consumption can disrupt BA homeostasis by inducing aberrant changes in the expression of genes involved in BA metabolism, causing dysbiosis of gut microbiota involved in BA metabolism, and modulating BA receptors (25). In addition to dietary influences, BA metabolism follows a distinct circadian pattern. An early study in healthy men revealed that serum unconjugated BAs exhibit diurnal variation, with most reaching peak concentrations between breakfast and dinner and remaining relatively stable from midnight until breakfast (26). BA synthesis itself displays a clear bimodal circadian rhythm, with the first peak around 13:00 and the second around 21:00, followed by a gradual decline from nighttime to the next morning (27). A recent clinical study further demonstrated that both conjugated and unconjugated BAs in healthy men follow a significant 24−hour rhythm. Notably, under sleep deprivation conditions, six BAs (including CA, GCA, TCA and LCA) retained their rhythmicity, suggesting that the circadian rhythm of BAs is primarily driven by environmental factors rather than the endogenous clock (28). Beyond diet and circadian regulation, the gut microbiota profoundly shapes the BA profile by modulating the enzymatic reactions involved in BA synthesis and altering the efficiency of deconjugation and dehydroxylation (1). The rate of enterohepatic circulation directly influences the size of the BA pool. In addition, BA homeostasis is finely tuned by BA receptors, a topic that will be discussed in detail below.

## 3 Bile acids act as versatile signaling molecules

BAs primarily mediate their biological effects by activating specific nuclear and membrane-bound receptors. Among these, FXR and TGR5 serve as the core receptors mediating BA functions. In addition to these core receptors, BAs also exert their biological actions through other receptor pathways, including the vitamin D receptor (VDR) (29), pregnane X receptor (PXR) (30), constitutive androstane receptor (CAR) (31), sphingosine-1-phosphate receptor 2 (S1PR2) (32), muscarinic receptors (MR) (33), and Mas-related G protein-coupled receptor family member E (MRGPRE) (34).

### 3.1 Structure-Activity Relationships of Bile Acids in Receptor Signaling

The functional diversity of BA signaling is intrinsically linked to their chemical structures. The number, position, and stereochemistry of hydroxyl groups on the steroidal core represent the primary determinants of FXR versus TGR5 selectivity. For instance, CDCA (with 3α,7α‑dihydroxy groups) exhibits high affinity for FXR; introduction of a hydroxy group at the C11β position yields highly selective FXR agonists (e.g., TC‑100), whereas C16α‑hydroxylation (e.g., pythocholic acid) confers selective TGR5 agonism without activating FXR (35, 36). C‑23(S) methylation similarly imparts marked TGR5 selectivity, while 7α‑methylation of UDCA enhances TGR5 activation but reduces FXR activity (37). Microbial transformation, particularly 7α‑dehydroxylation, reshapes the BA pool and altering the balance of FXR versus TGR5 agonists. Emerging modifications—including succinylation, acylation, and amine conjugation—further expand the functional repertoire of BAs (**Table 1**). Collectively, these structure‑activity relationships establish that BA receptor signaling is not a monolithic property but a finely tunable response shaped by the specific chemical modifications present in the circulating BA pool.

**Table 1:** Bile Acid Modifications and Their Functional Implications.

Modification Type	Representative Forms	Producing Bacteria	Receptor/Signaling Impact	Effect	Ref.
Acylation	3-O-acyl-CAs	*Christensenella minuta*	Inhibiting intestinal FXR	Ameliorating dysregulated glucose and lipid metabolism	(4)
Succinylation	3-succinylated CA	*Bacteroides uniformis*	FXR/TGR5-independent	Alleviating MASH	(5)
Alternative amino acid conjugation	Phenylalanine, tyrosine, leucine conjugates	Multiple gut bacteria	Potentially activating FXR	Representing a major mechanism of host-microbe metabolic interaction	(6)
Long-chain fatty acid conjugation	3β-acyl esters (isoLCA, isoDCA with C16/C18)	Not yet characterized	Not yet characterized	Accounting for ~29.7% of fecal BA pool	(7)
Polyamine conjugation	CA/DCA with spermine, spermidine	Not yet characterized	Not yet characterized	Antimicrobial activity	(8)

CA, cholic acid; DCA, deoxycholic acid; FXR, Farnesoid X receptor; MASH, metabolic dysfunction-associated steatohepatitis; TGR5, Takeda G-protein receptor 5

### 3.2 The FXR-centered classical nuclear receptor pathway

**Figure 3:**
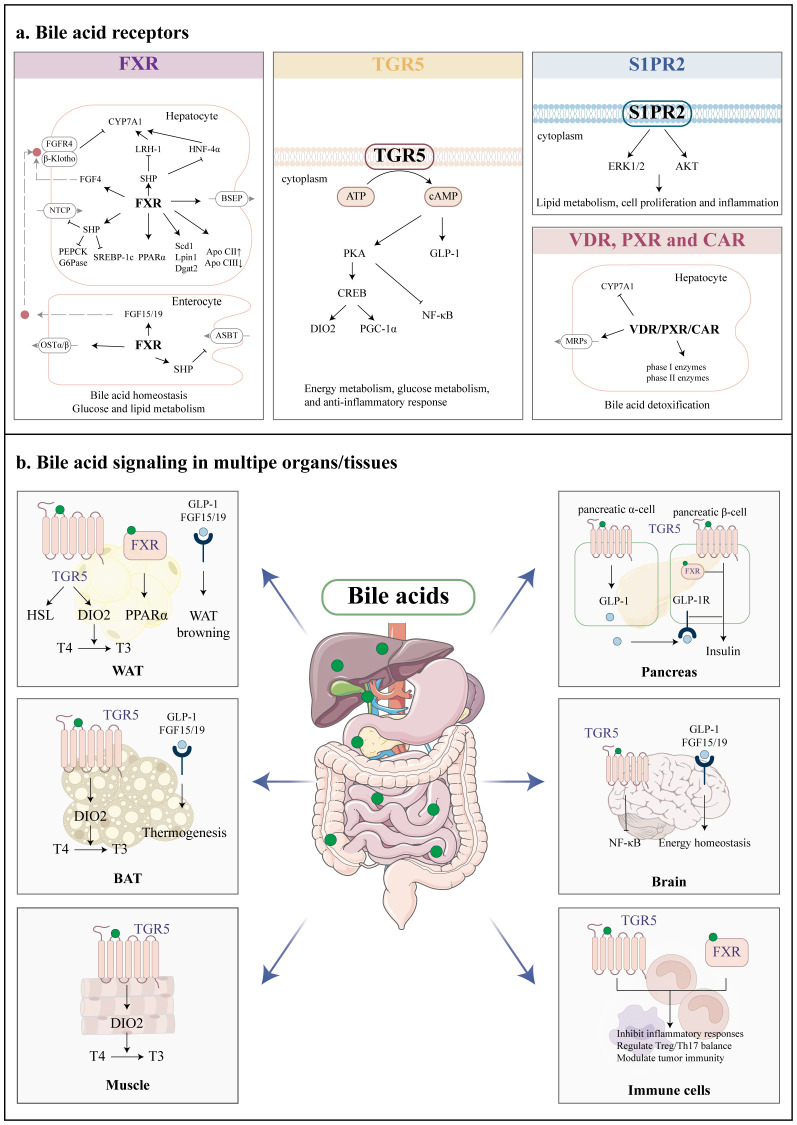
Bile acid receptors and their physiological function in multiple organs and tissues. **(a)** The signaling pathways and function of major BA receptors, involved in processes including BA homeostasis, glucose/lipid metabolism, energy metabolism and anti-inflammatory response. **(b)** The physiological function of BA signaling in multiple organs and tissues. ASBT, apical sodium-dependent bile acid transporter; BAs, bile acids; BAT, brown adipose tissue; BSEP, bile salt export pump; CAR, constitutive androstane receptor; CYP7A1, cholesterol 7α-hydroxylase; DIO2, type 2 iodothyronine deiodinase; FXR, Farnesoid X receptor; FGF, fibroblast growth factor; GLP-1, glucagon-like peptide-1; G6Pase, glucose-6-phosphatase; HNF-4α, hepatic nuclear factor 4α; HSL, hormone-sensitive lipase; LRH, liver receptor homolog; MRP, multidrug resistance-associated protein; NTCP, Na^+^-dependent taurocholate co-transporting polypeptide; OST, organic solute transporter; PEPCK, phosphoenolpyruvate carboxykinase; PGC-1α, peroxisome proliferator-activated receptor γ coactivator 1α; PPARα, peroxisome proliferator activated receptor α; PXR, pregnane X receptor; S1PR2, sphingosine-1-phosphate receptor 2; SHP, small heterodimer partner; SREBP-1c, sterol regulatory element-binding protein-1c; TGR5, G protein-coupled receptor Takeda G-protein receptor 5; VDR, vitamin D receptor; WAT, white adipose tissue.

FXR is mainly expressed in the metabolic and excretory organs, including liver, intestine, kidney, adipose tissue and adrenal cortex (21). Most natural BA species function as natural agonists for FXR, with the activation potency ranked as follows: CDCA > LCA > DCA > CA (38). FXR plays a central role in BA homeostasis (**Figure 2**) (39). Elevated BAs in hepatocytes activate FXR, leading to small heterodimer partner-1 (SHP-1) −mediated inhibition of HNF-4α and LRH-1, which in turn suppresses CYP7A1 and CYP8B1 expression (40, 41). Under cholestatic stress, hepatic FXR also directly activates fibroblast growth factor 4 (FGF4) transcription, which inhibits BA synthesis by activating signaling through the FGFR4-β-Klotho complex (42). Additionally, hepatic FXR promotes BA excretion by upregulating the canalicular transporter BSEP while inhibiting the sinusoidal uptake transporter NTCP via SHP, reducing BA reuptake from portal vein blood (43, 44). In the distal ileum, elevated BA levels also activate intestinal FXR, inducing secretion of FGF15/19 (FGF15 in mice; FGF19 in humans) (45, 46). This endocrine factor enters the bloodstream, circulates to the liver, and activates FGFR4-β-Klotho receptors on hepatocytes to suppresses CYP7A1 expression through MAPK-dependent pathways (45, 47). Intestinal FXR activation also increases expression of intestinal I-BABP and OSTα/OSTβ, enhancing BA transport from the intestine to the portal vein. Notably, during the early post-meal stage, the ileal FGF15/19 signaling pathway lags behind the liver’s role in BA synthesis due to time and spatial constraints (42). Beyond its function in BA homeostasis, FXR also plays a significant role in regulating lipid homeostasis, glucose homeostasis, and inflammatory response (**Table 2**; **Figure 3a**) (39).

**Table 2:** Regulation of FXR on BA, lipid, glucose homeostasis and inflammatory response.

Target	Tissues	Mechanism	Effect	Ref.
Bile acid homeostasis				
CYP7B1	Liver	FXR→SHP→LRH-1→inhibiting CYP7B1 expression	Inhibiting BA synthesis	(48)
CYP8B1	Liver	FXR→SHP→LRH-1→inhibiting CYP8B1 expression	Inhibiting BA synthesis	(48)
FGF4	Liver	FXR→FGF4→FGFR4-β-Klotho complex→LRH-1→inhibiting CYP7B1 and CYP8B1 expression	Inhibiting BA synthesis	(42)
BSEP	Liver	Direct induction by FXR	Facilitating BA excretion	(49)
NTCP	Liver	FXR→SHP→inhibiting NTCP expression	Reducing BA reuptake	(49)
FGF15/19	Intestine	FXR→FGF15/19→hepatic FGFR4-β-Klotho complex→ERK/JNK pathways→inhibiting CYP7B1 expression	Inhibiting BA synthesis	(45, 47)
I-BABP	Intestine	Direct induction by FXR	BA binding protein	(50)
OSTα/β	Intestine	Direct induction by FXR	Enhancing intestinal BA transport to the portal vein	(49)
Lipid homeostasis				
SREBP-1c	Liver	FXR→SHP→SREBP-1c	Inhibiting hepatic lipogenesis	(51)
Scd1, Lpin1, Dgat2	Liver	Direct induction by FXR	Inhibiting hepatic lipogenesis	(52)
PPARα	Liver	Direct induction by FXR	Promoting fatty acid β oxidation	(53)
VLDL receptor	Liver	FXR→SHP→VLDL receptor	Reducing plasma TG levels	(49)
ApoCII	Liver	Direct induction by FXR	Reducing plasma TG levels	(49)
ApoCIII	Liver	Direct inhibition by FXR	Reducing plasma TG levels	(49)
p-JNK	Liver	FXR→p-JNK→HNF4α→SR-BI	Reducing plasma HDL cholesterol levels	(54)
SMPD3	Intestine	FXR→SMPD3→ceramide	Increasing cholesterol levels	(55)
Glucose homeostasis				
PEPCK, G6Pase	Liver Intestine	FXR→SHP→HNF-4/FOXO1 FXR→FGF15/19→CREB→PGC-1α	Inhibiting gluconeogenesis	(50)
glycogen synthase	Intestine	FXR→FGF15/19→GSK 3α/3β→activating glycogen synthase	Promoting glycogenesis	(20)
Inflammatory response				
Inflammatory cytokines	Immune cells	FXR→inhibiting NK-κB pathways	Inhibition inflammatory reaction	(56)
NLRP3 inflammasome	Immune cells	Direct inhibition by FXR	Inhibition inflammatory reaction	(57)

ApoC II, apolipoprotein C-II; ApoCIII, apolipoprotein C-III; BA, bile acid; BSEP, bile salt export pump; CREB, cAMP response element-binding protein; CYP7A1, cholesterol 7α-hydroxylase; CYP8B1, cholesterol 12α-hydroxylase; Dgat2, diacylglycerol O-acyltransferase 2; FGF, fibroblast growth factor; FGFR4, fibroblast growth factor receptor 4; FOXO1, forkhead box protein O1; FXR, Farnesoid X receptor; G6Pase, glucose-6-phosphatase; GSK, glycogen synthase kinase; HNF4α, hepatocyte nuclear factor 4 α; I-BABP, ileal bile acid binding protein; Lpin1, phosphatidate phosphatase LPIN1; LRH-1, liver receptor homolog-1; NTCP, Na^+^-dependent taurocholate co-transporting polypeptide; OST, organic solute transporter; PEPCK, phosphoenolpyruvate carboxykinase; PGC-1α, peroxisome proliferator-activated receptor γ coactivator 1α; PPARα, peroxisome proliferator activated receptor α; Scd1, stearoyl-CoA desaturase 1; SHP, small heterodimer partner; SMPD3, sphingomyelin phosphodiesterase 3; SR-BI, scavenger receptor class B type I; SREBP-1c, element binding protein 1c; TG, triglycerides; VLDL, very low density lipoprotein.

### 3.3 The TGR5-centered classical membrane receptor pathway

TGR5 is widely expressed in adipose tissue, intestines, gallbladder, and brain (58). Its primary natural ligands, ranked by potency, are LCA, DCA, CDCA, CA, and UDCA (38). TGR5 mediates a significant portion of BAs’ effects on whole-body metabolism (**Figure 3a**) (59). Its activation induces the expression of type 2 iodothyronine deiodinase (DIO2) in adipocytes and skeletal muscle cells via cAMP-PKA-CREB signaling pathway (60). Then, DIO2 catalyzes the conversion of thyroxine (T4) to the biologically active triiodothyronine (T3), upregulating genes involved in energy expenditure, such as peroxisome proliferator-activated receptor γ coactivator 1 (PGC-1), uncoupling protein 1 (UCP-1), and UCP-3 (61). In intestinal L cells, TGR5 activation enhances mitochondrial oxidative phosphorylation and increases the ATP/ADP ratio, which further promotes glucagon-like peptide-1 (GLP-1) secretion (62). In immune cells, TGR5-cAMP-PKA signaling regulates inflammatory cytokine secretion and immune response by inhibiting NF-κB activation (63). TGR5 also exhibits anti-inflammatory effects through the TGR5-STAT3-A20 signaling in sepsis (64). However, under chronic injury (e.g., long-term gastric acid), the TGR5-STAT3-KLF5 pathway becomes chronically activated, promoting progression of precancerous lesions (65). In summary, TGR5 modulates diverse biological processes, including energy metabolism, glucose metabolism, and anti-inflammatory response (**Figure 3a**).

### 3.4 FXR/TGR5 independent BA-signaling pathway

In addition to the canonical FXR and TGR5 pathways, BAs also signal through other GPCRs (e.g., S1PR2, MR) and nuclear receptors (e.g., VDR, PXR, CAR) (**Figure 3a**). S1PR2, broadly expressed in liver cells, bile duct cells, stellate cells and intestinal epithelial cells, has been identified as a direct molecular target of conjugated BAs (e.g., TCA) (60). Activation of S1PR2 regulates hepatic lipid accumulation by upregulating proteins involved in fatty acid transport (e.g., ApoB 100) and fatty acid oxidation (e.g., CPT-1α) via SphK2/S1P axis (60). Adversely, under cholestatic conditions, its activation stimulates hepatic stellate cell proliferation and extracellular matrix protein secretion, contributing to the development of liver fibrosis (66). Additionally, S1PR2-mediated activation of ERK1/2 and AKT signaling enhances the malignant phenotype of cholangiocarcinoma cells (67). The M2 and M3 subtypes of muscarinic receptors (MR) are also regulated by BAs and aberrant overexpression of these receptors is linked to the initiation and progression of digestive system tumors, including colorectal, gastric, and cholangiocarcinoma (33, 68, 69).

BAs also serve as ligands for other nuclear receptors, including VDR, PXR, and CAR (29–31). VDR is widely expressed in bones, intestine, kidney, and liver (29). Beyond its classical role in calcium and phosphorus metabolism, VDR is also activated by LCA to inhibit BA synthesis via blocking hepatic nuclear factor 4α (HNF-4α)-mediated activation of CYP7A1 (29, 70). Conversely, VDR activation directly inhibits SHP expression, promoting cholesterol conversion to BAs (71). This suggests VDR-mediated regulation of CYP7A1 is bidirectional, dependent on ligand dose, cell type, and physiological status. LCA, DCA, and their conjugates serve as endogenous agonists of PXR (72). PXR activation inhibits CYP7A1 transcription by competing with HNF-4α (73). Additionally, PXR activation upregulates hepatic phase I enzymes (e.g., CYP3A11, CYP2B6), phase II enzymes (e.g., UDP-glucuronosyl transferase 1A1), and efflux transporters (e.g., MRP2/3), exerting detoxifying effects (74). CAR is mainly expressed in hepatic tissues, where it plays a critical role in regulating drug metabolism and detoxification (75). For example, in familial intrahepatic cholestasis type 5 (PFIC5), CAR activation alleviates liver injury by upregulating detoxifying enzymes (e.g., CYP3A, CYP2B) and promoting BA excretion via MRP2/3 and BSEP (75).

## 4 Bile acid-mediated inter-organ communication network

BAs act as key mediators of a sophisticated inter-organ communication network, integrating the function of the liver, intestine, pancreas, adipose tissue, muscle, brain and immune cells to regulate systemic homeostasis (**Figure 3b**).

### 4.1 Core axis: gut-liver axis

BAs are critical molecular mediators of the gut-liver axis (GLA). Liver-derived BAs reach the ileum, inducing fibroblast growth factor 15/19 (FGF15 in mice; FGF19 in humans) synthesis and secretion by FXR (45, 46). FGF15/19 travels to the liver via the portal vein, activating fibroblast growth factor receptor 4 (FGFR4) and co-receptor β-Klotho on hepatocytes to suppress BA synthesis, thus completing a gut-liver feedback loop (50). The gut microbiota and GLA form an integrated system. The microbiota shapes the BA pool through enzymatic transformations; BSH deconjugate primary BAs into free forms, which are then converted by specific anaerobes into secondary BAs (13, 50). Gut microbiota-derived BAs exert dual effects on liver health: excessive secondary BAs (e.g., LCA) induce hepatocyte damage via cytotoxicity (76), while other secondary BAs, such as 3-succinylated cholic acid (3-sucCA), alleviates metabolic dysfunction-associated steatohepatitis (MASH) by enriching beneficial gut bacteria (5). Pathologically, intestinal dysbiosis exacerbates liver disease progression via the GLA. In intrahepatic cholestasis of pregnancy (ICP), elevated *Bacteroides fragilis* levels suppress FXR signaling through high BSH activity, leading to increased BA synthesis and hepatic BA accumulation (77). Elevated conjugated BAs further promote hepatic inflammation by activating S1PR2 signaling (77).

### 4.2 Gut-metabolic organs axis

BAs function as key metabolic molecules, establishing an endocrine network that connects the intestine with peripheral metabolic organs, including the pancreas, adipose tissue, and muscle. In pancreatic β-cells, both the activation of TGR5 and FXR promote glucose-stimulated insulin secretion by elevating cytosolic calcium levels (10). Under hyperglycemic conditions, TGR5 activation upregulates the expression of PC1/3 via the cAMP-PKA-CREB signaling, promoting a shift from secreting glucagon to secreting GLP-1 in pancreatic α-cells (78). Furthermore, the GLP-1 secreted by intestinal L cells acts on pancreatic β-cells as an intestinal-derived signaling molecule to regulate insulin secretion (22). Interestingly, although secondary BAs alone do not increase glucagon secretion, under high-protein diet conditions, they interact with arginine to synergistically promote glucagon production (79). Abnormalities in BA-mediated insulin signaling are key mechanisms in the pathogenesis of diabetes (80). type 2 diabetes mellitus (T2DM) is closely associated with disruptions in BA metabolism, including enhanced synthesis and altered composition (60). For instance, the weakened inhibitory effect of insulin on *CYP8B1* gene expression leads to an increase in the ratio of 12α-hydroxylated to non-12α-hydroxylated BAs in T2DM (81). These altered BAs exert a negative impact on glucose metabolism disorders by inhibiting the TGR5/FXR-mediated pathway (82).

BAs also influence adipose tissue and muscle to modulate energy expenditure. FXR activation not only promotes adipocyte differentiation, but also enhances peripheral insulin sensitivity in adipose tissue and skeletal muscle (60, 83). Similarly, TGR5 significantly influences energy metabolism: its activation induces white adipose tissue browning, promotes brown adipose tissue thermogenesis and muscle energy expenditure (62, 84). Notably, BAs regulate energy homeostasis through the gut-adipose tissue axis. In the intestinal tract, particularly in the ileum, the activation of FXR and TGR5 triggers the release of intestinal signaling molecules (e.g., GLP-1, FGF15/19) into the circulation, which then modulate adipocyte function by inducing white adipose tissue browning and increasing brown adipose tissue thermogenic capacity (85, 86). Disruptions in BA metabolism cause imbalanced energy metabolism and metabolic diseases. A meta-analysis shows obesity is closely linked to BA metabolic disorders, including reduced FGF19, increased BA synthesis, and elevated fecal BA excretion (87). In high-fat diet-induced obese mice, altered BA composition correlates with downregulated PGC-1α and UCP-1 expression in brown adipose tissue (88). Conversely, administration of the TGR5 agonist alleviates obesity by promoting adipose tissue lipolysis and thermogenesis (89). Collectively, BAs regulate systemic energy metabolism balance through a signaling network centered on FXR and TGR5.

### 4.3 Gut-brain axis

The gut-brain axis is a key signaling pathway linking the gastrointestinal tract and central nervous system. Key brain regions like the cerebral cortex, hippocampus, and hypothalamus express BA receptors, including TGR5, FXR, and S1PR2, with TGR5 playing a central role (10, 90). The gut-brain axis regulates systemic energy homeostasis and glucose metabolism. Post-meal increases in BA levels activate TGR5 in the nucleus of the solitary tract, enhancing leptin-STAT3 signaling to reduce food intake (91). Additionally, gut-derived FGF15/19 activates the β-klotho/FGFR complex in the hypothalamus, boosting energy expenditure and improving glucose tolerance (92). Another critical gut-brain signal, GLP-1, reaches the brain via circulation or vagal nerves after nutrient-induced secretion, regulating appetite and energy metabolism (93). Beyond metabolism, GLP-1 provides neuroprotection, anti-inflammation, and cognitive enhancement through its widespread CNS receptor distribution (94).

### 4.4 Gut-immune axis

Research shows that both innate and adaptive immune cells express BA receptors (63). Activating FXR or TGR5 receptors reduces systemic inflammation by suppressing NF-κB pathways and NLRP3 inflammasome activation (56, 57, 63). TGR5 further balances classical (M1) and alternative (M2) macrophage phenotypes through immunoregulatory pathways, promoting a mixed phenotype with dominant immunosuppressive M2-like traits (95). Microbiota-derived molecules (e.g., SCFAs, secondary BAs, and tryptophan metabolites) play key roles in regulating T cell function (96). For example, 3-oxo-LCA binds to RORγt to block pro-inflammatory TH17 cell differentiation, while isoalloLCA promotes regulatory T (Treg) cell development by increasing mitochondrial reactive oxygen species (97). In the intestinal lamina propria, specialized RORγ^+^ Tregs, dependent on gut commensals, are vital for controlling intestinal inflammation and maintaining homeostasis (98). BAs regulate RORγ^+^ Treg differentiation and function via VDR (99). Pathological disruptions in BA metabolism, however, lead to immune imbalances. Multiple autoimmune diseases, such as autoimmune uveitis and multiple sclerosis, are linked to reduced serum total and secondary BAs due to gut microbiota dysbiosis impairing BA biotransformation (63, 100). This BA metabolic dysfunction weakens FXR/TGR5 signaling, contributing to immune regulatory imbalance.

BAs exhibit a dual role in tumor immunity. Research has indicated that microbiota-derived BAs, such as 3-oxo-Δ5-LCA, 3-oxo-Δ4-LCA, and 3-oxo-Δ4,6-LCA, serve as antagonists of the androgen receptor, inhibiting the proliferation of AR‑expressing prostate cancer cell lines (LNCaP, C4‑2) (101). However, in patients with hepatocellular carcinoma (HCC) or colorectal cancer (CRC), elevated levels of conjugated BAs are strongly correlated with tumor progression (102, 103). Elevated BAs promote tumor immune escape by activating FXR or TGR5, which induces secretion of immunosuppressive cytokines (e.g., TGF-β and IL-10) to inhibit the activity of T cells and NK cells (104). Additionally, in lung cancer models, DCA promotes immune evasion by activating TGR5 signaling, which drives STAT3 phosphorylation and upregulates PD_L1 expression (105).

## 5 Communication network disorders and therapeutic interventions

**Figure 4:**
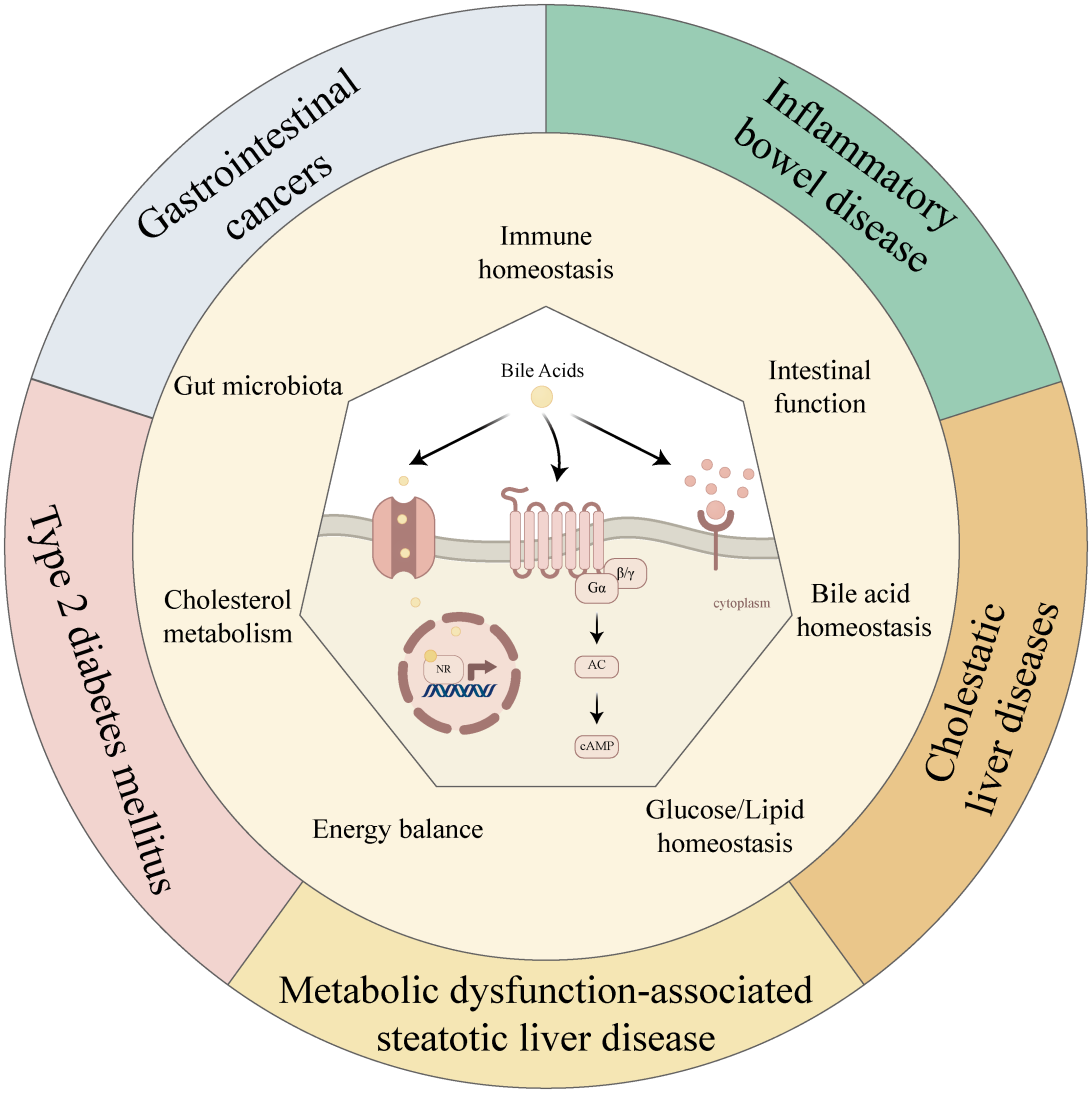
Bile acid signaling in health and disease. BAs and their signaling pathways function as central regulators in sustaining physiological health. The biochemistry, physiology, and pharmacology of BAs underpin protective mechanisms against disease, whereas dysregulated BA signaling or metabolism contributes to pathogenesis. BA-based therapeutics hold substantial clinical promise; continued research in this area may lead to novel interventions for hepatic, gastrointestinal and metabolic diseases. BAs, bile acids.

Fluctuations in BA levels or dysregulation of the signaling pathways disrupt inter-organ communication, potentially leading to metabolic disorders and immune dysregulation (10). Meanwhile, gut microbiota dysbiosis contributes to these disorders by altering the composition and size of the BA pool. Consequently, targeting BAs for therapeutic intervention offers novel strategies for diagnosing and treating complex diseases (**Figure 4**).

### 5.1 Metabolic dysfunction-associated steatotic liver disease

MASLD/metabolic dysfunction-associated steatohepatitis (MASH) is associated with BA pool remodeling, characterized by suppression of the alternative BA synthetic pathway and reduced non-12-hydroxylated BAs (106). Mechanistically, CYP7B1, the key enzyme of this pathway, is consistently downregulated in MASLD mouse models and human cohorts, leading to decreased non-12-OH BAs, which subsequently drive LXR/PPAR‑mediated hepatic lipogenesis and exacerbates liver injury (106). Circulating BA changes (e.g., elevated total BAs and altered secondary BA) correlate with histological features of metabolic dysfunction-associated steatohepatitis (MASH), such as steatosis, lobular inflammation, ballooning, and fibrosis (20, 107, 108). More importantly, hepatic FXR expression is consistently diminished in MASLD/MASH, compromising the FXR‑mediated feedback inhibition of BA synthesis and further exacerbating the metabolic imbalance (109). Gut microbiota dysbiosis in MASLD patients promotes harmful BA production, exemplified by increased Bacteroidetes (carrying DCA-synthesis genes) and decreased protective Ruminococcaceae during disease progression (110).

BAs and their receptors have emerged as therapeutic targets for MASLD (111). The FXR agonist obeticholic acid (OCA) improves biochemical and histological parameters in MASH and attenuates fibrosis (112), but its clinical use is limited by pruritus and elevated LDL-C (113). Notably, serum hyodeoxycholic acid (HDCA) is reduced in MASLD patients, and HDCA supplementation alleviates disease by inhibiting intestinal FXR, upregulating hepatic CYP7B1, and enriching the beneficial bacterium *Parabacteroides distasonis*, which enhances lipid metabolism through the fatty acid-liver peroxisome proliferator activated receptor α (PPARα) signaling pathway (114). Administration of tauroursodeoxycholic acid (TUDCA) ameliorates MASLD by inhibiting intestinal inflammation and improving intestinal barrier function (107). Microbiota−targeted interventions (probiotics, prebiotics, fecal microbiota transplantation [FMT]) improve BA metabolism and reduce BMI, cholesterol, LDL−C, and triglycerides in MASLD patients (115). The gut commensal *Bacteroides uniformis* produces the secondary BA 3−succinylated cholic acid (3−SucCA), which enhances intestinal barrier function and reverses MASH by promoting *Akkermansia muciniphila* (5). Furthermore, bariatric surgery promotes metabolic benefits independent of weight loss in MASLD patients by inducing gut microbiota recolonization and reversing the primary-to-secondary BA ratio (116). Additionally, BA synthetic enzymes represent druggable targets (117). CYP7A1 governs overall BA pool size and cholesterol catabolism. In Cyp7a1⁻/⁻ mice, a methionine/choline-deficient (MCD) diet significantly accelerates hepatic free cholesterol accumulation, oxidative stress, apoptosis, and inflammation, and promotes liver fibrosis; conversely, adenovirus-mediated overexpression of Cyp7a1 effectively reduces hepatic inflammation (117). Hu et al. show that celastrol (CEL), a natural bioactive compound, significantly ameliorates MASH in Western diet- and HFD-induced mouse models by activating FXR/LXR signaling, which upregulates CYP7B1 expression and restores the suppressed alternative BA synthetic pathway (118). This positions CYP7B1 and its upstream FXR/LXR regulators as actionable targets beyond conventional FXR agonism. Similarly, CYP27A1 expression is consistently downregulated in MASLD/MASH models, suggesting CYP27A1 may also be a promising therapeutic target. (119, 120). Collectively, restoring BA homeostasis through receptor targeting, microbiota modulation or synthetic enzymes holds promise for ameliorating steatosis, inflammation, and fibrosis in MASLD.

### 5.2 Type 2 diabetes mellitus

BA-mediated signaling pathways involving FXR and TGR5 have been identified as promising therapeutic targets for obesity and T2DM (121). Experiments show that supplementation with GUDCA leads to increased levels of tauro-LCA and increased abundance of *Bacteroides vulgatus*, which collectively activate TGR5 in adipose tissue and upregulate the expression of the uncoupling protein 1 (UCP-1), thereby exerting an anti-T2DM effect (12). GUDCA has also been identified as an endogenous intestinal FXR antagonist, and metformin—a first-line antidiabetic drug—exerts its glucose-lowering effects partly through the Bacteroides fragilis–GUDCA–intestinal FXR axis (122). Furthermore, research has identified a distinct class of BAs in pigs, called hyocholic acids (HCAs), that act on intestinal endocrine L cells to simultaneously activate the TGR5 receptor and inhibit FXR signaling, which enhances the secretion of GLP-1 and thus reduces blood glucose (123). In summary, BA signaling, particularly through receptors like FXR and TGR5 and mediated by specific acids like GUDCA and HCAs, represents a promising therapeutic target for T2DM by modulating gut microbiota and improving glucose metabolism. Beyond the aforementioned endogenous BA species, emerging evidence indicates that current incretin/glucagon-based therapies—including GLP‑1 receptor agonists (GLP‑1RAs), dual GLP‑1R/GIPR agonists (e.g., tirzepatide), and GCGR agonists—actively modulate BA signaling, and this interplay may contribute to their metabolic benefits. GLP‑1RAs (e.g., liraglutide) have been shown to reduce total BA levels and downregulate intestinal FXR signaling, accompanied by decreased expression of BA transporters such as ASBT and I‑BABP, as well as suppressed hepatic Cyp7a1 expression via enhanced FGF15‑mediated feedback (124). In diabetic mouse models, the dual GLP‑1R/GIPR agonist tirzepatide reshapes the BA profile by increasing the ratio of FXR antagonists (e.g., β‑MCA, GUDCA, UDCA) to FXR agonists, while simultaneously reducing intestinal FXR expression (125). This shift toward FXR antagonism suggests a mechanism distinct from that of pure GLP‑1RAs. In contrast, GCGR agonism (e.g., IUB288) increases plasma BA levels, and its weight‑reducing and energy‑expenditure‑promoting effects are largely dependent on intact hepatic FXR signaling; in liver‑specific FXR-/- mice, these metabolic benefits are abolished (126). Collectively, these findings reveal a bidirectional crosstalk between incretin/glucagon receptor agonists and BA signaling, and suggest that BA profiles may serve as potential biomarkers for predicting individual responses to these therapies, although clinical validation is warranted.

### 5.3 Cholestatic liver disease

CLDs are characterized by the dysfunction of BA secretion, which consequently lead to progressive liver injury, inflammation, and fibrosis (127). Currently, UDCA remains the first-line therapeutic agent for most CLDs, such as primary biliary cholangitis (PBC) (128). However, its therapeutic efficacy is limited to the early disease stage, and over 40% of patients exhibit a suboptimal response (129). Thus, there is an urgent need to explore combination therapy or alternative approaches. Targeting BA transporters, including intestinal IBAT inhibitors and hepatic NTCP inhibitors, exerts therapeutic effects (130). For instance, the IBAT inhibitors odevixibat and maralixibat have been approved for the treatment of pediatric cholestatic disorders, such as progressive PFIC (131). Notably, over 40 -50% of patients with PFIC treated with IBAT inhibitors exhibit insufficient clinical responses, suggesting that simply blocking intestinal BA reabsorption may not achieve adequate efficacy in some patients (132). Concurrently, the long-term benefits of IBAT inhibitors in adult chronic CLDs remain unclear (132). Regarding safety, long-term IBAT inhibition is associated with concerns including diarrhea, fat-soluble vitamin deficiency, and potential hepatotoxicity (133, 134). In response to hepatotoxicity risks, the FDA updated the labeling for odevixibat and maralixibat in June 2025, establishing previous or current hepatic decompensation (e.g., variceal hemorrhage, ascites, hepatic encephalopathy) as contraindications and strengthening warnings regarding liver injury. Collectively, these findings underscore that the expansion of IBAT inhibitor indications to broader populations requires careful deliberation regarding their long-term safety and uncertain efficacy. Nuclear receptor-targeted drugs have emerged as a research focus in recent years. An international multicenter randomized double-blind parallel phase 3 trial demonstrated that obeticholic acid markedly ameliorated cholestatic biochemical parameters in PBC patients with suboptimal response or intolerance to UDCA, yet the drug was associated with a higher rate of adverse events including pruritus and increased LDL-C versus placebo (135). These adverse effects ultimately contribute to the withdrawal of OCA from the European market. From a BA pool perspective, OCA inhibits CYP7A1-driven BA synthesis, causing hepatic cholesterol accumulation that suppresses SREBP-2 activity and LDL receptor expression, thereby reducing hepatic LDL clearance and elevating circulating LDL-C; this lipid effect can be reversed by atorvastatin co-administration (136). The pruritus appears to be a class effect of FXR agonists, possibly mediated by OCA's concurrent activation of TGR5 (GPBAR1) and MRGPRX4 (137). Recently, Yang et al. demonstrate that cholestatic pruritus is linked to the 3-hydroxyl group (3-OH) on BAs and develop a 3-OH-deficient OCA derivative that retains FXR agonistic activity without inducing pruritus, as a novel candidate for liver disease therapy (138). Additionally, non-steroidal FXR agonists (e.g., cilofexor, tropifexor) improve alkaline phosphatase (ALP) levels and cause less severe pruritus, holding promise as new alternative agents (128). PPAR agonists exert therapeutic effects by regulating BA metabolism. Mechanistic studies have revealed that hepatocyte PPARα controls BA homeostasis by downregulating hepatic BA uptake transporters (e.g., NTCP, OATP1, OATP4), decreasing BSEP expression, and upregulating basolateral efflux transporters MRP3 and MRP4 (139). PPARδ agonism, on the other hand, suppresses BA synthesis by reducing hepatocyte CYP7A1 through the FGF21 signaling pathway (140). Novel PPAR agonists, such as seladelpar (a PPARδ agonist) and elafibranor (a PPARα/δ agonist), significantly reduce ALP levels and have recently been approved as second-line treatments for PBC (141, 142). Currently, the treatment of CLD is shifting from traditional drugs to precise targeted interventions. The development of new drugs such as novel FXR agonists, PPAR agonists, and microbial therapies has demonstrated significant therapeutic potential in clinical trials.

### 5.4 Inflammatory bowel disease

IBD is a group of immune-mediated chronic inflammatory bowel conditions, primarily comprising two major subtypes: Crohn’s disease (CD) and ulcerative colitis (UC) (143). Current therapies (aminosalicylates, corticosteroids, immunomodulators, biologics) mainly alleviate symptoms without halting disease progression (144). Given the close regulatory interplay between BAs, gut microbiota, and intestinal inflammation, targeting the microbiota-BA-inflammation axis may be a novel therapeutic direction. Preclinically, supplementation with *Bifidobacterium adolescentis* or *Zymomonas mobilis* shows efficacy in colitis models (145, 146). FMT has also been shown to effectively alleviate clinical symptoms in active UC patients (143). Meanwhile, the supplementary secondary BAs significantly attenuates intestinal inflammation through TGR5 signaling (147). Experiments have demonstrated the FXR agonist OCA effectively inhibits intestinal inflammation and enhance the intestinal barrier function (148). Together, these strategies—probiotics, FMT, and BA receptor modulators—hold therapeutic promise for IBD.

### 5.5 Gastrointestinal cancers

Beyond its role in chronic intestinal inflammation, dysregulated BA metabolism also directly fuels gastrointestinal carcinogenesis. Dysregulated BA metabolism—such as elevated serum total and secondary BAs—promotes gastrointestinal cancer (e.g., CRC) by inducing DNA damage, suppressing anti-tumor immunity, and activating pro-carcinogenic pathways (95, 149, 150). FXR exerts tumor-suppressive functions by disrupting the β−catenin/TCF4 complex and antagonizing Wnt/β−catenin signaling (151). FXR agonist OCA has been found to reverse tissue fibrosis and inhibit tumor growth, although its anti-tumor effects are more pronounced in the early stages (152). Probiotic interventions also show promise: *Weissella cibaria* upregulates FXR and inhibits NF-κB, supporting its use in CRC (153). Metabolic surgery (e.g., Roux−en−Y gastric bypass) may prevent CRC metastasis by altering enterohepatic BA circulation (154). Although BA−targeted therapies remain experimental, modulating gut microbiota and BA metabolism to affect proliferation and immune−related pathways holds significant potential for suppressing tumor growth and metastasis.

## 6 Conclusions and perspectives

The dynamic composition of the BA pool, shaped by host synthesis and microbial modification, allows fine-tuned regulation of physiological processes via distinct receptor pathways. In this review, we highlight that BAs are not merely biomarkers of disease but active drivers of metabolic and immune homeostasis—and when dysregulated, key contributors to systemic pathologies (60). These insights lay a mechanistic groundwork for developing BA-based therapeutics, pointing toward strategies that target specific nodes of the BA signaling network to treat metabolic and immune-mediated diseases.

However, the development of effective and safe therapies targeting BA signaling faces several challenges. First, the complexity of the BA pool and the tissue-specific distribution of BA receptors, particularly in humans, remain incompletely characterized (10). Second, non-specific FXR activation causes significant side effects (e.g., pruritus, dyslipidemia), whereas tissue-restricted FXR modulation is technically difficult and lacks clear criteria for target organ selection. Third, activation of the same receptor in different tissues may lead to distinct or even opposing effects due to differences in downstream signaling, receptor subtypes, or local microenvironment (10). Fourth, species differences exist between humans and mice in BA composition, which complicate the interpretation of preclinical data and pose a major barrier to clinical translation. Meanwhile, advancing research has challenged conventional understanding. For example, contrary to its long-standing characterization as an intestinal FXR antagonist, recent evidence identifies UDCA as an ileal agonist (155).

Recent advances in elucidating the mechanistic roles of BAs in whole-body organs and tissues have established BA signaling as a promising therapeutic strategy. Despite considerable challenges, the therapeutic potential of BA-based interventions remains substantial, and continued research is likely to accelerate their clinical translation.

## Glossary

ASBT: apical sodium-dependent bile acid transporter
BAAT: bile acid CoA:amino acid N-acyltransferase
BAs: bile acids
BSEP: bile salt export pump
BSH: bile salt hydrolase
CA: cholic acid
CAR: constitutive androstane receptor
CDCA: chenodeoxycholic acid
MR: muscarinic receptor
CRC: colorectal cancer
CYP7A1: cholesterol 7α-hydroxylase
CYP8B1: cholesterol 12α-hydroxylase
CYP27A1: cholesterol 27-hydroxylase
CYP7B1: cholesterol 7α-hydroxylase
CYP2C70: cholesterol 6β-hydroxylase
DCA: deoxycholic acid
DIO2: type 2 iodothyronine deiodinase
FGF: fibroblast growth factor
FGFR4: fibroblast growth factor receptor 4
FMT: fecal microbiota transplantation
FXR: Farnesoid X receptor
GLA: gut-liver axis
GLP-1: glucagon-like peptide-1
HDCA: hyodeoxycholic acid
HNF-4α: hepatic nuclear factor 4α
I-BABP: ileal bile acid binding protein
IBD: inflammatory bowel disease
LCA: lithocholic acid
MASLD: metabolic dysfunction-associated steatotic liver disease
MASH: metabolic dysfunction-associated steatohepatitis
MCA: muricholic acid
MRP: multidrug resistance-associated protein
NTCP: Na^+^-dependent taurocholate co-transporting polypeptide
OATP: organic anion transporting polypeptide
OCA: obeticholic acid
OST: organic solute transporter
PGC: peroxisome proliferator-activated receptor γcoactivator
PPARα: peroxisome proliferator activated receptor α
PXR: pregnane X receptor
SCFAs: short-chain fatty acids
S1PR2: sphingosine-1-phosphate receptor 2
SHP: small heterodimer partner
3-sucCA: 3-succinylated cholic acid
T2DM: type 2 diabetes mellitus
TGR5: Takeda G-protein receptor 5
TUDCA: tauroursodeoxycholic acid
UCP: uncoupling protein
UDCA: ursodeoxycholic acid
VDR: vitamin D receptor.
